# Supplementary material for: Modeling glioblastoma heterogeneity as a dynamic network of cell states
Source: Mol Syst Biol. 2021 Sep 16;17(9):e10105. doi: 10.15252/msb.202010105 (PMC8444284; doi:10.15252/msb.202010105)
Supplement: Supplementary file 6 — Source Data for Figure 5 [file MSB-17-e10105-s004.zip › Figure5A_sourcedata/GSEA_3071/hallmarks_stateI.GseaPreranked.1623742531165/HALLMARK_UV_RESPONSE_DN.html]

Details for gene set HALLMARK\_UV\_RESPONSE\_DN[GSEA]

|  || Dataset | state13071 |
| Phenotype | NoPhenotypeAvailable |
| Upregulated in class | na\_pos |
| GeneSet | HALLMARK\_UV\_RESPONSE\_DN |
| Enrichment Score (ES) | 0.36138245 |
| Normalized Enrichment Score (NES) | 1.9238418 |
| Nominal p-value | 0.005882353 |
| FDR q-value | 0.005642585 |
| FWER p-Value | 0.036 |
Table: GSEA Results Summary

  

Fig 1: Enrichment plot: HALLMARK\_UV\_RESPONSE\_DN      
 Profile of the Running ES Score & Positions of GeneSet Members on the Rank Ordered List

  

| PROBE | GENE SYMBOL | GENE\_TITLE | RANK IN GENE LIST | RANK METRIC SCORE | RUNNING ES | CORE ENRICHMENT || 1 | COL3A1 |  |  | 3 | 2.646 | 0.0636 | Yes |
| 2 | COL1A1 |  |  | 6 | 2.008 | 0.1120 | Yes |
| 3 | PDGFRB |  |  | 9 | 1.893 | 0.1576 | Yes |
| 4 | COL11A1 |  |  | 37 | 1.468 | 0.1856 | Yes |
| 5 | COL5A2 |  |  | 48 | 1.373 | 0.2161 | Yes |
| 6 | TFPI |  |  | 64 | 1.241 | 0.2421 | Yes |
| 7 | NRP1 |  |  | 81 | 1.180 | 0.2662 | Yes |
| 8 | SNAI2 |  |  | 142 | 0.940 | 0.2717 | Yes |
| 9 | EFEMP1 |  |  | 169 | 0.876 | 0.2856 | Yes |
| 10 | ANXA4 |  |  | 190 | 0.847 | 0.3004 | Yes |
| 11 | IGFBP5 |  |  | 198 | 0.833 | 0.3187 | Yes |
| 12 | SYNE1 |  |  | 214 | 0.800 | 0.3339 | Yes |
| 13 | ATP2B1 |  |  | 270 | 0.713 | 0.3353 | Yes |
| 14 | DAB2 |  |  | 277 | 0.702 | 0.3507 | Yes |
| 15 | ATP2B4 |  |  | 330 | 0.644 | 0.3513 | Yes |
| 16 | ZMIZ1 |  |  | 365 | 0.624 | 0.3567 | Yes |
| 17 | MGLL |  |  | 406 | 0.581 | 0.3593 | Yes |
| 18 | NOTCH2 |  |  | 446 | 0.551 | 0.3614 | Yes |
| 19 | TJP1 |  |  | 557 | 0.477 | 0.3411 | No |
| 20 | SMAD7 |  |  | 597 | 0.454 | 0.3409 | No |
| 21 | LAMC1 |  |  | 640 | 0.439 | 0.3394 | No |
| 22 | ID1 |  |  | 682 | 0.420 | 0.3378 | No |
| 23 | BHLHE40 |  |  | 720 | 0.405 | 0.3370 | No |
| 24 | GJA1 |  |  | 727 | 0.403 | 0.3450 | No |
| 25 | DUSP1 |  |  | 787 | 0.386 | 0.3374 | No |
| 26 | LPAR1 |  |  | 829 | 0.372 | 0.3345 | No |
| 27 | ACVR2A |  |  | 934 | 0.343 | 0.3128 | No |
| 28 | MET |  |  | 974 | 0.333 | 0.3096 | No |
| 29 | PMP22 |  |  | 1069 | 0.312 | 0.2900 | No |
| 30 | CDK13 |  |  | 1089 | 0.309 | 0.2920 | No |
| 31 | ITGB3 |  |  | 1102 | 0.306 | 0.2960 | No |
| 32 | NR3C1 |  |  | 1162 | 0.297 | 0.2861 | No |
| 33 | NEK7 |  |  | 1176 | 0.293 | 0.2895 | No |
| 34 | MAP1B |  |  | 1198 | 0.289 | 0.2905 | No |
| 35 | SIPA1L1 |  |  | 1254 | 0.276 | 0.2813 | No |
| 36 | ADD3 |  |  | 1261 | 0.275 | 0.2862 | No |
| 37 | CAV1 |  |  | 1341 | 0.261 | 0.2697 | No |
| 38 | ABCC1 |  |  | 1359 | 0.258 | 0.2711 | No |
| 39 | SLC7A1 |  |  | 1389 | 0.254 | 0.2688 | No |
| 40 | TGFBR2 |  |  | 1410 | 0.251 | 0.2692 | No |
| 41 | CITED2 |  |  | 1782 | -0.291 | 0.1688 | No |
| 42 | DDAH1 |  |  | 1791 | -0.292 | 0.1736 | No |
| 43 | PIK3R3 |  |  | 1797 | -0.293 | 0.1793 | No |
| 44 | KCNMA1 |  |  | 1849 | -0.299 | 0.1718 | No |
| 45 | CDON |  |  | 1852 | -0.300 | 0.1785 | No |
| 46 | SFMBT1 |  |  | 2061 | -0.327 | 0.1262 | No |
| 47 | ARHGEF9 |  |  | 2327 | -0.379 | 0.0587 | No |
| 48 | NR1D2 |  |  | 2602 | -0.440 | -0.0100 | No |
| 49 | MAGI2 |  |  | 2732 | -0.480 | -0.0357 | No |
| 50 | MT1E |  |  | 2982 | -0.591 | -0.0934 | No |
| 51 | RBPMS |  |  | 3015 | -0.610 | -0.0878 | No |
| 52 | DLG1 |  |  | 3043 | -0.628 | -0.0803 | No |
| 53 | SERPINE1 |  |  | 3050 | -0.634 | -0.0666 | No |
| 54 | CDKN1B |  |  | 3065 | -0.646 | -0.0549 | No |
| 55 | AMPH |  |  | 3066 | -0.648 | -0.0391 | No |
| 56 | SRI |  |  | 3177 | -0.757 | -0.0525 | No |
| 57 | INPP4B |  |  | 3263 | -0.871 | -0.0559 | No |
| 58 | PLPP3 |  |  | 3335 | -0.988 | -0.0524 | No |
| 59 | NFIB |  |  | 3388 | -1.166 | -0.0390 | No |
| 60 | MMP16 |  |  | 3423 | -1.321 | -0.0167 | No |
| 61 | CELF2 |  |  | 3464 | -1.718 | 0.0136 | No |
Table: GSEA details [plain text format]

  

Fig 2: HALLMARK\_UV\_RESPONSE\_DN: Random ES distribution      
 Gene set null distribution of ES for **HALLMARK\_UV\_RESPONSE\_DN**
